# Global proteomic profiling in multistep hepatocarcinogenesis and identification of PARP1 as a novel molecular marker in hepatocellular carcinoma

**DOI:** 10.18632/oncotarget.7316

**Published:** 2016-02-11

**Authors:** Xiao Xu, Zhikun Liu, Jianguo Wang, Haiyang Xie, Jie Li, Jili Cao, Lin Zhou, Shusen Zheng

**Affiliations:** ^1^ Division of Hepatobiliary and Pancreatic Surgery, First Affiliated Hospital, Zhejiang University School of Medicine, Hangzhou, China; ^2^ Key Laboratory of Combined Multi-Organ Transplantation, Ministry of Public Health, Hangzhou, China; ^3^ Collaborative Innovation Center for Diagnosis and Treatment of Infectious Diseases, Hangzhou, China

**Keywords:** hepatocellular carcinoma, hepatocarcinogenesis, biomarker, PARP1

## Abstract

The more accurate biomarkers have long been desired for hepatocellular carcinoma (HCC). Here, we characterized global large-scale proteomics of multistep hepatocarcinogenesis in an attempt to identify novel biomarkers for HCC. Quantitative data of 37874 sequences and 3017 proteins during hepatocarcinogenesis were obtained in cohort 1 of 75 samples (5 pooled groups: normal livers, hepatitis livers, cirrhotic livers, peritumoral livers, and HCC tissues) by iTRAQ 2D LC-MS/MS. The diagnostic performance of the top six most upregulated proteins in HCC group and HSP70 as reference were subsequently validated in cohort 2 of 114 samples (hepatocarcinogenesis from normal livers to HCC) using immunohistochemistry. Of seven candidate protein markers, PARP1, GS and NDRG1 showed the optimal diagnostic performance for HCC. PARP1, as a novel marker, showed comparable diagnostic performance to that of classic markers GS and NDRG1 in HCC (AUCs = 0.872, 0.856 and 0.792, respectively). A significant higher AUC of 0.945 was achieved when three markers combined. For diagnosis of HCC, the sensitivity and specificity were 88.2% and 81.0% when at least two of the markers were positive. Similar diagnostic values of PARP1, GS and NDRG1 were confirmed by immunohistochemistry in cohort 3 of 180 HCC patients. Further analysis indicated that PARP1 and NDRG1 were associated with some clinicopathological features, and the independent prognostic factors for HCC patients. Overall, global large-scale proteomics on spectrum of multistep hepatocarcinogenesis are obtained. PARP1 is a novel promising diagnostic/prognostic marker for HCC, and the three-marker panel (PARP1, GS and NDRG1) with excellent diagnostic performance for HCC was established.

## INTRODUCTION

Hepatocellular carcinoma (HCC) is a worldwide prevalent and deadly neoplasia, which occurs almost in the background of cirrhotic liver as a result of chronical hepatitis virus infection. The prevalence of HBV carriage is reported to be 350 million people worldwide [1]. China has a high HBV prevalence, with approximately 93 million individuals with chronic HBV [2]. It has been reported that 15–20% of chronic hepatitis B patients progress to cirrhosis within 5 years and that the annual incidence of HCC is 2.8% [3]. Hepatocarcinogenesis is a typical multistage process characterized by chronic viral infection, liver cirrhosis, and HCC [4, 5].

Despite remarkable advances in diagnostic and therapeutic techniques [6, 7], the molecular pathogenesis is extremely complex and heterogeneous even intratumor [8], long-term survival rates remain poor. The best method of achieving long-term survival is diagnosing the disease at an asymptomatic stage when potentially curative treatments are feasible [9]. Surveillance of patients at the highest risk for developing HCC, i.e., patients with cirrhosis, is a critical strategy that can potentially decrease the cancer-related mortality rate [10]. Therefore, the more accurate markers have long been desired to discriminate HCCs from dysplastic nodules (DN) and liver cirrhosis, or to have good prognostic performance for patients with HCC [11]. The global large-scale protein profiles of multistep hepatocarcinogenesis are an important step toward the identification of new diagnostic and/or prognostic biomarkers and therapeutic targets. The most protein markers of HCC arise from the various established methods, including indirect gene expression analysis (gene arrays) and direct proteomics [12]. The establishment of isobaric tags for relative and absolute quantitation (iTRAQ) and two-dimensional liquid chromatography−tandem mass spectrometry (2D LC−MS/MS) for large-scale analysis of protein expression is a new tool for markers, which has not been used in the multistage hepatocarcinomagenisis. iTRAQ 2D LC−MS/MS has made it possible to seek novel molecular markers in more large-scale proteomics for diagnosis, outcome prediction, and identifying molecules involved in carcinogenesis in a process of tumor development.

In this study, iTRAQ-2D LC-MS/MS was used to quantitatively analyze the protein alternations of multistep HBV-related hepatocarcinogenesis. We compared protein profiles in a series of 5 pooled samples: healthy subjects, patients with HBV hepatitis, patients with HBV cirrhosis, patients with HCC and their peritumoral tissues. The discovery of the molecular profiles will help to peep into histologic process of hepatocarcinogenesis. From the seven candidate markers, we identified PARP1 as a new promising diagnostic/prognostic biomarker for HCC and established a three-marker panel (PARP1, GS and NDRG1) greatly improving the diagnostic accuracy of HCC in liver nodules. This is the first report concerning the clinical utility of PARP1 for diagnosis and prognosis in patients with HCC.

## RESULTS

### Protein expression profiles in multistep hepatocarcinogenesis by iTRAQ

**Figure 1:**
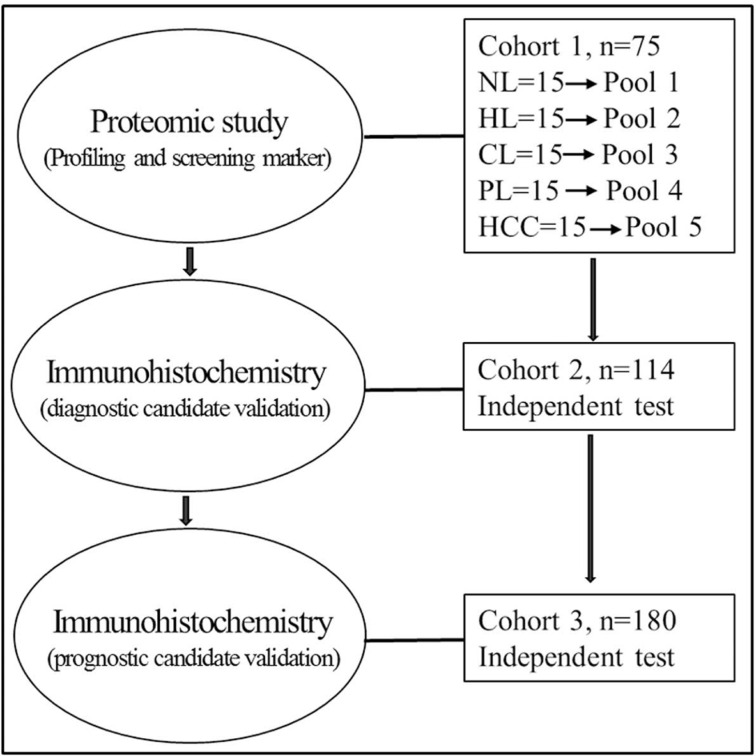
Overview of the study design for marker discovery and verification Normal livers, NL, hepatitis livers, HL, cirrhotic livers, CL, peritumoral livers, PL, HCC livers, HCC.

**Figure 2:**
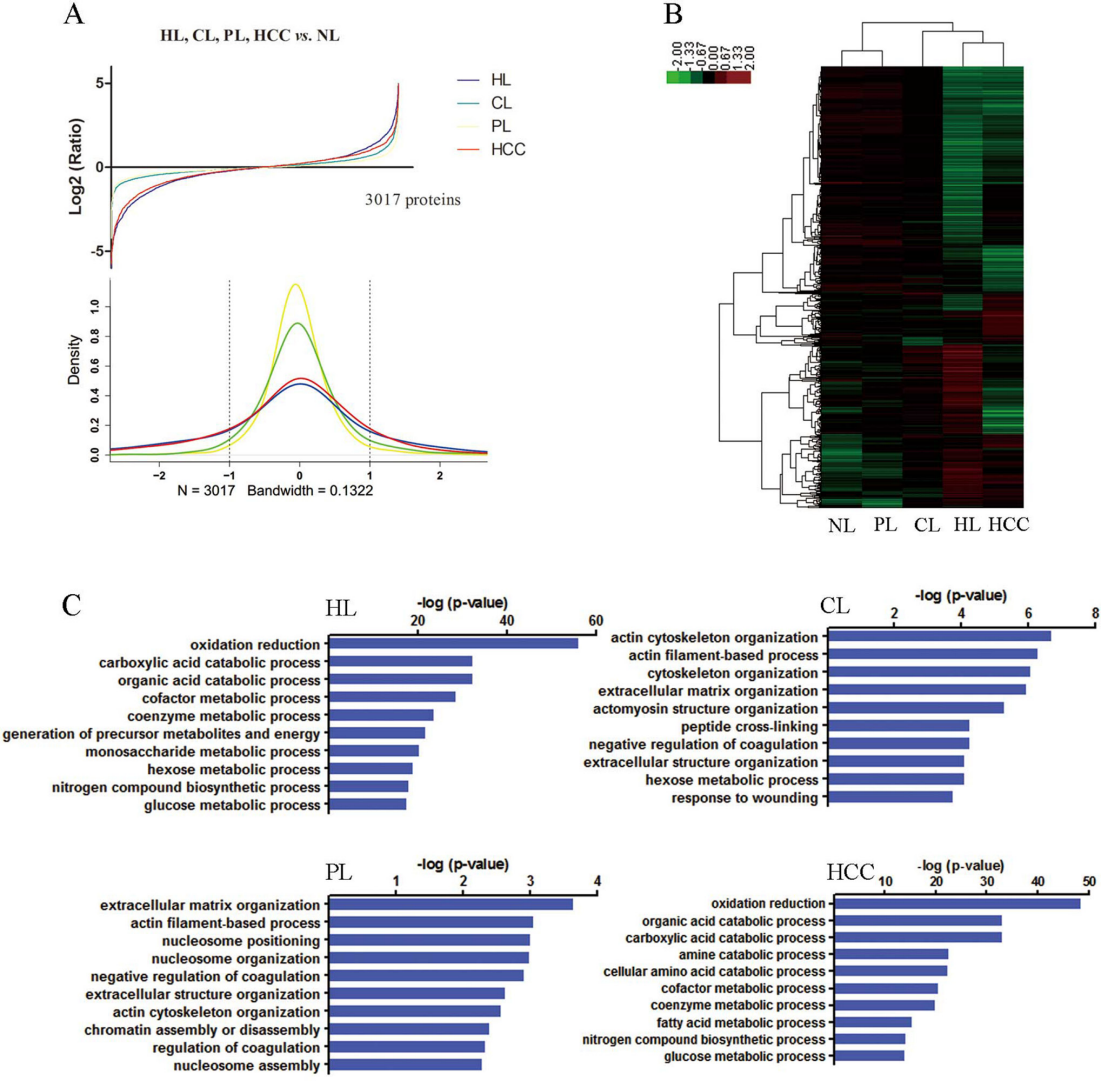
Global protein expression patterns on the spectrum of multistep hepatocarcinogenesis and biological functional annotation (**A**) The protein expression distributions (upper) and densities representing the distribution (lower) of all proteins in HL (blue), CL (green), PL (yellow), and HCC (red). The dotted line represents threshold of 2 fold-change. (**B**) Hierarchical clustering of all identified proteins. HL and PL clustered together. HL and HCC comprised a major sample cluster. (**C**) Gene ontology function analysis of the differentially expressed proteins in HL, CL, PL and HCC groups compared with NL, the top 10 biological process were presented.

To identify protein expression patterns on the spectrum of hepatocarcinogenesis, we performed comparative protein profiling in 5 pooled samples (Figure 1, Table S1): NLs, HLs, CLs, PLs and HCCs using iTRAQ 2D LC−MS/MS. In order to reduce false positive results, a strict cutoff for protein identification was applied with the unused ProtScore > 1.3 and at least one peptide with 95% confidence limit [13]. We obtained quantitative data on 37874 sequences and 3017 proteins during hepatocarcinogenesis (Tables S2, S3). Figure 2A showed fold changes of all identified protein in groups of HLs, CLs, PLs, and HCCs relative to NLs. The number of differentially expressed proteins (the fold change cutoff ratio < 0.5 or > 2.0) were highest in HLs followed by HCCs, and the lowest in PLs. We compared global protein expression patterns among different groups. Hierarchical clustering of all identified proteins was performed (Figure 2B). NL and PL groups clustered well together. HL and HCC groups had similar proteomic patterns and comprised a major sample cluster, CLs added to this group yielded another cluster. These data suggested that the difference between PLs and NLs is minimal, supporting the fact that malignant nodule can be removed with PLs left for patients. Functional annotations of these differentially expressed proteins (compared to NL group) were analyzed. The top 10 behaviors of the biological process were shown in Figure 2C. Biological processes responsible for production of extracellular matrix/components are significantly involved in CLs and PLs, but more obviously in CLs than in PLs. Although PLs are peritumoral tissues, PLs suffer from chronic hepatitis and proceed to cirrhosis. Biological processes about energy production (essential for cell division, e.g., carboxylic acid and glucose metabolic processes) as well as oxidation reduction were most markedly observed in HLs and HCCs.

### Screening and validation of diagnostic marker for HCC

**Figure 3:**
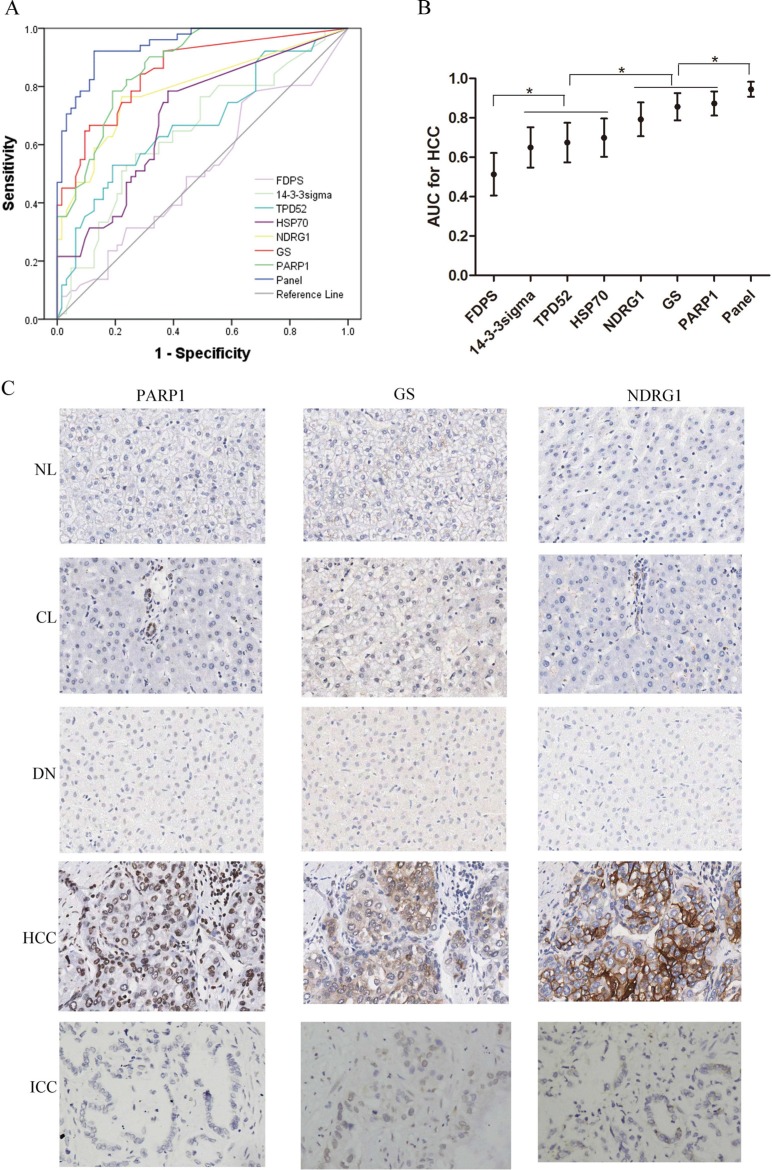
Diagnostic performance of FDPS, 14-3-3sigma, TPD52, HSP70, NDRG1, GS, and PARP1 for HCC in 40 nonmalignant nodules (7 NLs, 19 CLs, 14 DNs), 51 HCCs (24 grade 1-2, and 27 grade 3) and 23 ICCs (**A**) ROCs of these candidate markers and three-marker panel (GS, NDRG1 and PARP1) for diagnosis of HCC. (**B**) The AUCs with 95% CI. AUCs of NDRG1, GS, and PARP1 were significantly higher than that for 14-3-3sigma, TPD52, and HSP70. AUCs of 14-3-3sigma, TPD52, and HSP70 were significantly higher than that of FDPS. The AUC value of panel significantly increased to 0.945, when PARP1, GS and NDRG1were combined. *p < 0.01. (**C**) The expression patterns of PARP1, GS and NDRG1 examined by immunohistochemistry in a series of liver tissues (40×). NL, CL, NL and ICC tissues negatively expresses PARP1, GS and NDRG1, while the HCC tissues show positive staining of the three markers. PARP1, GS and NDRG1 immunoreactivity were primarily examined in the nucleus, nucleocytoplasm and cytoplasm/cytomembrane, respectively. NL, normal liver; CL, cirrhotic liver; DN, dysplastic nodule; ICC, intrahepatic cholangiocarcinoma.

To screen the diagnostic markers of HCC, we focused on the proteins which upregulates in HCCs. The top six highest proteins were 14-3-3sigma, N-myc downstream regulated 1 (NDRG1), tumor protein D52 (TPD52), farnesyl pyrophosphate synthase (FDPS), glutamine synthetase (GS), and poly [ADP-ribose] polymerase 1 (PARP1) (Table 1). To validate whether these proteins are exclusively overexpressed in HCC, we stained them with antibodies in 40 nonmalignant nodules (7 NLs, 19 CLs, 14 DNs), 51 HCCs (24 grade 1–2, and 27 grade 3) and 23 ICCs (Table S4). Notably, heat shock 70 kDa protein (HSP70) ranked No. 11 on the list, and has been repeatedly reported as the diagnostic marker for HCC [2, 14, 15]. Therefore, HSP70 was also stained for reference. ROC curves were constructed to evaluate the area under the curve (AUC) for these potential markers. The AUCs for FDPS, 14-3-3sigma, TPD52, HSP70, NDRG1, GS, and PARP1 were 0.513, 0.650, 0.674, 0.699, 0.792, 0.856, and 0.872, respectively, and all the AUCs were significant compared with Reference Line except that for FDPS. The PARP1, GS, and NDRG1 showed optimal diagnostic performance, whose AUCs were significantly higher than that for 14-3-3sigma, TPD52, and HSP70. (Figure 3A, 3B).

**Table 1:** Top 20 proteins with highest upregulation (HCC vs other groups)

Proteins	Unused ProtScore	Rank (121 : others)	log 114:113	log 115:113	log 119:113	log 121:113	Confident Peptides
14-3-3 protein sigma	4.4	1	0.452	0.2	0.224	1	569
protein NDRG1 isoform 1	9.32	2	−0.64	0.292	0.06	0.652	178
tumor protein D52 isoform 2	8.2	3	−0.088	0.068	−0.136	0.576	394
farnesyl pyrophosphate synthase isoform a	26.5	4	−0.972	−0.236	0.016	0.428	220
glutamine synthetase	29.37	5	−0.384	−0.604	−0.092	0.38	193
poly [ADP-ribose] polymerase 1	35.82	6	0.352	0.064	0.116	0.744	168
alpha-1-antitrypsin precursor	79.22	7	−0.536	0.016	−0.032	0.496	163
HLA class I histocompatibility antigen, A-1 alpha chain precursor	26.16	8	0.564	0.292	0.388	0.936	146
major histocompatibility complex, class I, B precursor	6.13	9	0.508	−0.212	−0.092	0.724	156
fibrinogen-like protein 1 precursor	5.02	10	−0.44	0.116	−0.26	0.472	167
heat shock 70 kDa protein 1A/1B	69.19	11	0.128	0.224	0.228	0.72	154
stathmin isoform a	7.29	12	0.084	0.088	0.024	0.604	128
apolipoprotein E precursor	45.08	13	0.084	0.068	0.024	0.596	97
tapasin isoform 2 precursor	5.69	14	0.268	0.296	0.232	0.756	177
flap endonuclease 1	4.05	15	0.308	0.088	−0.052	0.644	136
inter-alpha-trypsin inhibitor heavy chain H3 preproprotein	5.64	16	−0.108	−0.164	0.036	0.48	183
C4b-binding protein alpha chain precursor	19.14	17	−0.236	−0.128	−0.028	0.44	138
DNA/RNA-binding protein KIN17	2.02	18	0.108	−0.064	0.1	0.568	165
activated RNA polymerase II transcriptional coactivator p15	13.93	19	0.564	0.352	0.332	0.88	91
aldo-keto reductase family 1 member C3 isoform 2	16.38	20	−0.464	0.18	−0.004	0.508	145

Normal liver (NL), 113; hepatitis liver (HL), 114; cirrhotic liver (CL), 116; peri-HCC liver (PL), 119; hepatocellular carcinoma (HCC), 121.

PARP1, GS, and NDRG1 were further analyzed for their performance of diagnosis for HCC. Of the three biomarkers, PARP1 was firstly validated as a novel diagnostic marker for HCC, GS is the classic marker for HCC [2, 15, 16], and NDRG1 is identified in HCC previously by our center [17] and other groups [18, 19]. Next, we compared the diagnostic performance of PARP1 for HCC with that of GS and NDRG1. The results demonstrated comparable AUCs of the three markers. Strikingly, the AUC value significantly increased to 0.945, when PARP1, GS and NDRG1 were combined (*p* < 0.001, Figure 3A, 3B). The immunohistochemical features of three markers were shown in Figure 3C.

The optimal cut-points for positive expressions of the three markers were determined in ROC curve analysis with the points closest to the point with both maximum sensitivity and specificity. Thus, tumors designated positive for PARP1, GS, and NDRG1 were those with values above the value of 25%, 5% and 5%, respectively. Using these criteria, the results were summarized in Table 2. All NLs were negatively stained by each of the three markers. The number of immuno-positive cases for which there was at least one marker increased from 7/19 (36.8%) in the case of CLs to 8/14 (57.1%) for DNs and to 51/51 (100%) for HCCs. Immuno-positive cases for which there were at least two markers (regardless of their identity) were observed in 0/7 NLs, in 1/19 (5.3%) CLs, in 4/14 (28.65%) DNs, in 20/24 (83.3%) G1/G2 HCCs, in 25/27 (92.6%) G3 HCCs, and in 7/23 (30.4%) ICCs (Table 2). Further statistical analysis showed that when at least two positive marker was considered, the sensitivity, specificity, positive predictive value (PPV), negative predictive value (NPV), accuracy and Youden index for differentiating HCCs from non-HCC tissues were 88.2%, 81.0%, 78.9%, 89.5%, 84.2% and 0.69, respectively (Table 3).

**Table 2:** Immunohistochemical features of the different lesions with the 3-marker panel

	Nonmalignant tissue/nodule			Malignant nodule		
	NL (*n* = 7)	CL (*n* = 19)	DN (*n* = 14)	G1/G2 HCC (*n* = 24)	G3 HCC (*n* = 27)	ICC (*n* = 23)
All three positive	0	0	0	10	17	3
At least two positive	0	1	4	20	25	7
At least one positive	0	7	8	24	27	19
PARP1+/GS+	0	0	2	14	19	4
PARP1+/NDRG1+	0	0	1	14	19	4
GS+/NDRG1+	0	1	1	12	21	5
PARP1+	0	3	4	20	21	8
GS+	0	3	3	18	25	13
NDRG1+	0	2	5	16	23	7

NL, normal liver; CL, cirrhotic liver; DN, dysplastic nodule; HCC G1/G2, well/moderately differentiated hepatocellular carcinoma; HCC G3, poorly differentiated hepatocellular carcinoma; ICC, intrahepatic cholangiocarcinoma.

**Table 3:** Diagnostic accuracy of the 3 marker panel for HCC discrimination form non-HCC

	Non-HCC (*n* = 63)	HCC (*n* = 51)	Sensitivity	Specificity	PPV	NPV	Accuracy	Youden index
Three markers								
All three positive	3	27	52.9	95.2	90.0	71.4	76.3	48.2
At least two positive	12	45	88.2	81.0	78.9	89.5	84.2	69.2
At least one positive	34	51	100	46.0	60.0	100	70.2	46.0
Two markers								
PARP1+/GS+	6	33	64.7	90.5	84.6	76.0	78.9	55.2
PARP1+/NDRG1+	5	33	64.7	92.1	86.8	76.3	79.8	56.8
GS+/NDRG1+	7	33	64.7	88.9	82.5	75.7	78.1	53.6
One markers								
PARP1+	15	41	80.4	76.2	73.2	82.8	78.1	56.6
GS+	19	43	84.3	69.8	69.4	84.6	76.3	54.2
NDRG1+	14	39	76.5	77.8	73.6	80.3	77.2	54.2

### Association between PARP1, GS and NDRG1 expression and clinicopathological features and survival of HCC patients

To validate the reproducibility of these findings and determine the association with specific pathologic features of HCC and with survival, we further performed immunohistochemistry assay of PARP1, GS and NDRG1 in another independent cohort including 180 paired HCCs and PLs. Using the positive criteria described above, the positive expression of PARP1, GS and NDRG1 were examined in 156/180(86.7%), 160/180 (88.8%), 146/180(81.1%) of HCCs, respectively, 29/180(16.1%), 40/180(22.2%), 35/180(19.4%) of PLs, respectively. Correlation analysis showed that the expression of NDRG1 in HCCs was significantly associated with tumor size and differentiation, PARP1 with tumor size and stage (*p* < 0.05, Table 4). However, no significant associations in characteristics of tumor, such as tumor stage, tumor size, etc. were observed in the high versus low GS groups.

**Table 4:** Relationship between PARP1, GS and NDRG1 expression and clinic-pathological characteristics in 180 HCC patients

Variables	PAPR1			GS			NDRG1		
	High	Low	*p*	High	Low	*p*	High	Low	*p*
Age (years)			0.638			0.433			0.158
≤ 50	33	29		34	28		26	36	
> 50	57	61		56	62		64	54	
Sex			0.686			0.418			0.686
Female	13	16		17	12		16	13	
Male	77	74		73	78		74	78	
Cirrhosis			1.000			0.321			0.185
Yes	26	25		29	22		21	30	
No	64	65		61	68		69	60	
Tumor size (total diameter)			**0.001**			0.545			**0.015**
≤ 5 cm	26	49		40	35		29	46	
> 5 cm	64	41		50	55		61	44	
Tumor multiplicity			1.0			0.195			1.0
Single	78	77		81	74		77	78	
Multiple	12	13		9	16		13	12	
Differentiation			0.179			0.629			**0.017**
Well	9	14		10	13		6	17	
Moderate	46	52		52	46		48	50	
Poor	35	24		28	31		36	23	
TNM stage			**0.045**			0.195			0.092
I	7	16		9	14		7	16	
II	37	39		44	32		36	40	
III	42	29		34	37		40	31	
IV	4	6		3	7		7	3	

**Figure 4:**
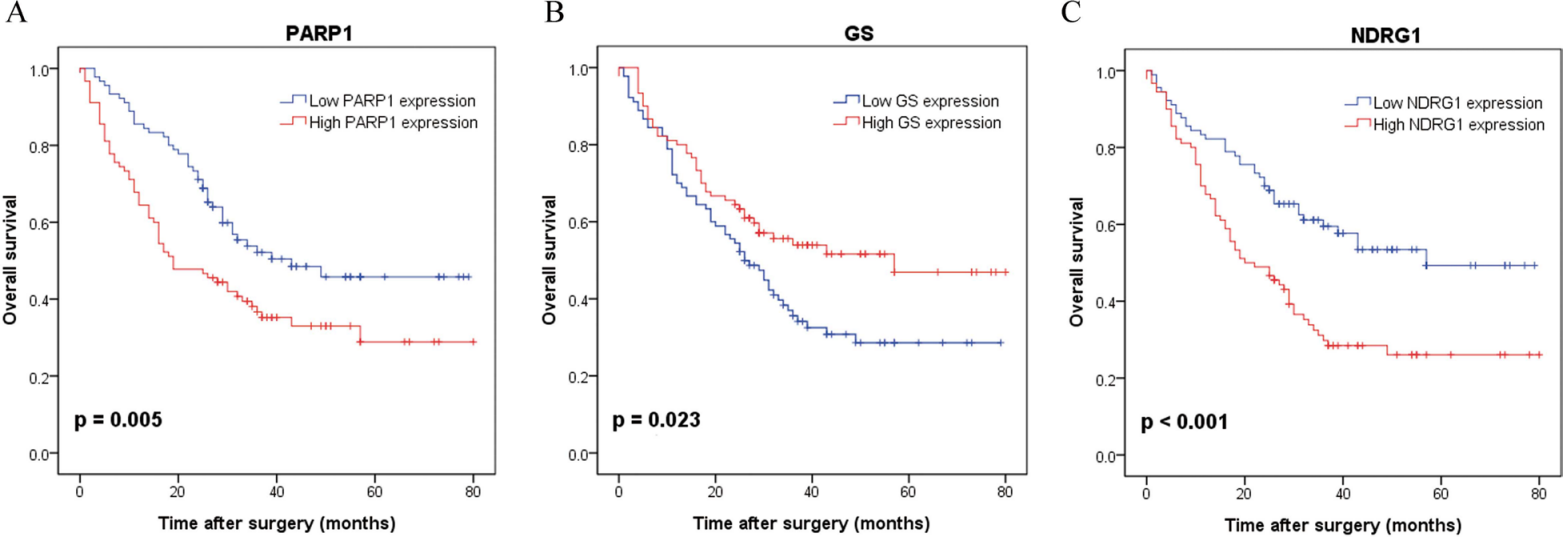
Kaplan-Meier survival curves with regard to overall survival according to PARP1, GS and NDRG1 protein expression in 180 patients with HCC (log-rank test) (**A**) Overall survival of patients in high expression of PARP1 significantly worse than that in low expression of PARP1 (*p* = 0.005). (**B**) Overall survival of patients in high expression of GS significantly better than that in low expression of GS (*p* = 0.023). (**C**) Overall survival of patients in high expression of NDRG1 significantly worse than that in low expression of NDRG1 (*p* < 0.001).

Kaplan-Meier analysis showed that patients with a high PARP1 expression had a significantly poorer prognosis than those with a low PARP1 expression (*p* = 0.005, Figure 4A). However, the patients with a high GS expression had a significantly better survival than those with a low GS expression (*p* = 0.023, Figure 4B). High NDRG1 expression was also associated with the poorer over survival as we reported previously (*p* < 0.001, Figure 4C). Further univariate and multivariate Cox regression analysis indicated that PARP1 and NDRG1 expression were the independent prognostic factors for poor survival of HCC patients (Table 5).

**Table 5:** Univariate and multivariate analysis of prognostic factors on overall survival

Variables	Univariate Analysis		Multivariate Analysis	
	Hazard Ratio (95% CI)	*p* Value	Hazard Ratio (95% CI)	*p* Value
Age (> 50 ys)	0.89 (0.59–1.33)	0.569		
Gender (male)	1.29 (0.73–2.26)	0.384		
Cirrhosis (yes)	0.84 (0.54–1.32)	0.448		
Tumor size (> 5 cm)	2.18 (1.43–3.34)	**< 0.001**	1.28 (0.78–2.10)	0.336
Tumor multiplicity (single)	0.52 (0.32–0.86)	**0.011**	0.60 (0.35–1.01)	0.054
Differentiation (mod./well vs poor)	0.61 (0.41–0.91)	**0.016**	0.75 (0.50–1.14)	0.178
TNM stage (I–II *vs* III–IV)	0.44 (0.29–0.65)	**< 0.001**	0.60 (0.38–0.96)	**0.035**
PRAP1 (high vs low)	1.75 (1.18–2.59)	**0.006**	1.52 (1.02–2.27)	**0.040**
GS (high *vs* low)	0.63 (0.43–0.94)	**0.023**	0.77 (0.51–1.16)	0.205
NDRG1 (high *vs* low)	2.07 (1.39–3.09)	**< 0.001**	1.70 (1.12–2.58)	**0.012**

## DISCUSSION

Timely and conclusive diagnostic reports are very important for the treatment of hepatocellular nodules. The effectively markers are required to make diagnosis and prognosis more objective and accurate. Although some of tissue biomarkers have been proposed and emphasized in clinical diagnostic/prognostic practice, these biomarkers should be validated in different aetiological/locational HCC, and compared with the newly discovered marker discovered by novel technology. iTRAQ was firstly used in the multistage hepatocarcinomagenisis and provide global protein profiles during hepatocarcinomagenisis. Of the top 6 highest proteins in HCC as well as classic maker HSP70, PARP1, GS and NDRG1 exhibited the best diagnostic performance for HCC, and additional predictive power could be achieved when using a 3 marker panel. PARP1, GS and NDRG1 were further verified in an independent cohort for diagnostic value and have different prognostic performance for HCC patients after operation.

iTRAQ-based proteomics gave an overview of the global protein alternation on the spectrum of multistage hepatocarcinogenesis in this study. Distinct protein markers with different stages of hepatocarcinogenesis could be recognized on the basis of these global protein expression data. Overall, the greatest difference was observed between NL and HL group, but not between NL and HCC group. And the PL cluster well together with NL, which support the clinical practice that malignant nodule can be removed with PL left for patients. Biological processes about energy production (essential for cell division, e.g., carboxylic acid and glucose metabolic processes) as well as oxidation reduction were most markedly observed in HLs and HCCs. Biological processes responsible for production of extracellular matrix/components are significantly involved in CLs and PLs.

We verified the diagnostic performance of these 6 upregulated proteins in HCCs, together with HSP70 in 40 non-HCC nodules, 51 HCCs, and 23 ICCs. Except FDPS, other 6 proteins have more or less diagnostic value for HCC. In consistent with Zhang, Y. [20], 14-3-3sigma is upregulated in HCC in our study, but the diagnostic value is not satisfactory (AUCs = 0.650). 14-3-3sigma is upregulated in gastric breast cancer [21] but downregulated in esophageal squamous cell carcinoma [22]. For TPD52, its overexpression has been described from a multitude of cancer types, including breast, prostate, ovarian, and has been linked to poor prognosis in breast and prostate cancer patients [23–26]. Little is known about TPD52 expression in liver cancer, we observed its upregulation in HCC but without good enough diagnostic value (AUCs = 0.674). About HSP70, it has been reported as the diagnostic marker for HCC lonely or combined with others and exhibited good accuracy [2, 15, 16], and ranked in the top 11. Therefore, we selected HSP70 as reference to evaluate diagnostic performance of focused proteins. However, HSP70 was not good enough diagnostic biomarker in our study (AUC = 0.699). This may be caused by different etiology of HCC. HCV-related HCC accounts for great majority in the previous publications [2, 15, 16], while our study included only HBV-related liver disease, which are major etiology of HCC in China. These data may indicate that HSP70 is an inferior diagnostic biomarker for HBV-related HCC. Further analysis showed that the diagnostic performance improve grealy, when PARP1, GS and NDRG1 were combined. To our attention, the three markers were stained with relatively high ratio in ICCs, if the ICCs were excluded from the cohorts, the accuracy of diagnosis HCCs would be much better. Especially, GS was stained in 13/23 (56.5%) ICCs. Of the three biomarker panel, GS is the classic marker for HCC [2, 15, 16]; NDRG1 is identified by our center [17] and other groups [18, 19]. PAPR1 increase the replication efficiency of HBV, inhibiting the DNA repair capacity, potentially contributing to the development of HCC [27]. PARP1 is firstly identified as diagnostic/prognostic marker for HCC in this study. Our findings not only confirm the value of GS and NDRG1 for the detection of HCC, but also establish a three-marker panel (PARP1, GS and NDRG1) with good diagnostic performanc of HBV-related HCC.

We further performed immunohistochemistry in an independent cohort of 180 HCC patients. High PARP1 expression was associated with larger tumor size and poorer survival. As we reported previously, NDRG1 was related with poorer survival. Interestingly, high GS staining was associated with better overall survival although GS failed to be an independent factor for overall survival. Indeed, Dal Bello *et al.* [28] showed that GS immunostaining correlates with reduced tumor-specific and lower overall mortality after radiofrequency ablation. However, GS expression is reported to be risk for HCC recurrence by Osada [29]. Further studies are needed to confirm the prognostic role of GS in the HCC patient.

In conclusion, this study has characterized the global protein expression profiles during multistage hepatocarcinogenesis, which provide a rich resource of proteins for further exploration about carcinogenesis. We identified PARP1 as a novel biomarker for HCC and demonstrated that a panel composed by NDRG1, GS, and PARP1 is very useful in distinguishing between CLs, DNs and HCCs. The diagnostic and prognostic values of PARP1 and its possible therapeutic applications are worth further investigation.

## MATERIALS AND METHODS

### Study cohorts and sample collection

The samples enrolled in this study were grouped into three independent cohorts based on their usage. The first cohort of 75 samples (Cohort1, Figure 1, Table S1) was used for biomarker discovery by proteomic profiling. These included 15 normal livers (NLs), 15 HBV hepatitis livers (HLs), 15 HBV cirrhotic livers (CLs), 15 HBV-related HCC livers (HCCs) and paired 15 peritumoral livers (PLs). The liver tissues of the cohort 1 after operation were immediately snap-frozen in liquid nitrogen and stored at −80°C until use. Formalin-fixed paraffin-embedded tissue samples were obtained from another two separate sets: cohort 2 of 114 subjects (Table S4), and cohort 3 of 180 patients (Table S5) and examined by immunohistochemistry. Cohort 2 consisted of a series of liver nodules (7 NLs, 19 CLs, 14 DNs, 51 HCCs (24 grade 1-2, and 27 grade 3) and 23 intrahepatic cholangiocarcinoma (ICCs). Cohort 3 contained 180 patients with HCC. The diagnosis of HCC was made by pathological examinations of the resected tissues after operation. No patients received preoperative treatments prior to the surgery. Usage of these samples was approved by Ethics Committee of the First Hospital of Zhejiang University, and all subjects in this study provided written informed consent. The overview of the clinical samples and the study design is illustrated in Figure 1.

### iTRAQ coupled with 2D LC−MS/MS analysis

To create quantitative protein expression profiles, an iTRAQ experiment was performed with 2D LC −MS/MS. In each group, proteins extracted from 15 different liver samples were equally mixed for proteomics analysis to improve profiling coverage and quantitative accuracy. [13, 30] The iTRAQ labeling was performed according to the manufacturer's instructions (Applied Biosystems, USA). Briefly, 100 μg of protein in each group was precipitated with ice-cold acetone overnight at at −20°C, and then the protein pellets were dissolved and digested using trypsin. The peptide mixtures from each group were labeled with the iTRAQ regents respectively as follows: NL, 113; HL, 114; CL, 115; PL, 119; HCC, 121 (Figure 1). The differentially iTRAQ-labeled peptides were mixed equally, desalted, and dried for subsequent analysis. The first dimension separation by High pH RP Chromatography was performed on an L-3000 HPLC System (Rigol) by using a C18 RP column (5 um, 250 mm, 4.6 mm i.d., Agela). In the second dimension, Fractions of peptides from the first dimension RPLC were separated by a Low pH RP column (3 um, 10 cm, 75 m i.d., C18) and then subjected to a Triple-TOF 5600 (Applied Biosystems) mass spectrometry for measurement. For protein identification and quantification, the complete set of raw data files (*.wiff) from Triple-TOF 5600 were searched by ProteinPilot version 4.2 using Paragon search engine against the human ref-sequence protein database. The ratios of the peak areas of the five iTRAQ reporter ions reflected the relative abundances of the peptides and the proteins in the above five groups. Cluster 3.0 software was used to investigate the hierarchical clustering of identified proteins. Java Treeview was used for visualization. The biological function of the identified proteins was analyzed on line DAVID.

### Immunohistochemistry assay

Expression of the interesting proteins was stained in paraffin-embedded liver samples from HCC patients in Cohort 2 and Cohort 3 (Tables S2, S3). Briefly, 4-μm sections were de-waxed and then treated with an antigen retrieval procedure and incubated in methanol containing 0.5% hydrogen peroxide for 20 min to block endogenous peroxidase. The sections were blocked in normal protein block serum solution, and then incubated with the primary antibody at 4°C overnight, and then washed by PBS buffer for 3 times (5 min of each) at room temperature. It further followed by incubating with HRP-conjugated secondary antibodies (BiotechInc, China) at room temperature for 1 hour. Finally, the sections were subjected to DAB staining and hematoxylin re-staining. A negative control was obtained by replacing the primary antibody with a normal murine or rabbit IgG. Immunoreactivity for proteins was scored using a semi-quantitative method by evaluating the number of positive cells over the total number of liver cells. Scores were assigned by using 5% increments (0%, 5%, 10%. 100%), as reported. [14] The results were independently assessed by two pathologists double-blindly, and concordance on agreed scores was achieved with a high k coefficient value (> 0.80). The antibodies and the dilution were detailed in the Table S6.

### Statistical analyses

Statistical analysis was performed using SPSS software version 18.0 (SPSS, Chicago, IL, USA). Qualitative variables were analyzed by the Fisher's exact test and Pearson's chi-squared test, while quantitative variables were analyzed by Student's *t* test. Receiver operating characteristic (ROC) curves were used to assess the diagnostic value of candidate proteins. The statistical significance of the correlation between biomarker expression and disease-specific survival was estimated by the log-rank test. Cox proportional hazards regression was carried out to identify the independent factors which significantly impact survival. All statistical tests were two-sided, and a *p*-value < 0.05 was considered statistically significant.

## SUPPLEMENTARY MATERIALS TABLES







## References

[1] (2012). EASL clinical practice guidelines: Management of chronic hepatitis B virus infection. J Hepatol.

[2] (2013). http://www.nhfpc.gov.cn/jkj/s3582/201307/518216575e544109b2caca07fca3b430.shtml.

[3] Kobashi H, Miyake Y, Ikeda F, Yasunaka T, Nishino K, Moriya A, Kubota J, Nakamura S, Takaki A, Nouso K, Yamada G, Yamamoto K (2011). Long-term outcome and hepatocellular carcinoma development in chronic hepatitis B or cirrhosis patients after nucleoside analog treatment with entecavir or lamivudine. Hepatol Res.

[4] Kim SW, Yang HG, Kang MC, Lee S, Namkoong H, Lee SW, Sung YC (2014). KIAA1114, a full-length protein encoded by the trophinin gene, is a novel surface marker for isolating tumor-initiating cells of multiple hepatocellular carcinoma subtypes. Oncotarget.

[5] Zavattari P, Perra A, Menegon S, Kowalik MA, Petrelli A, Angioni MM, Follenzi A, Quagliata L, Ledda-Columbano GM, Terracciano L, Giordano S, Columbano A (2015). Nrf2, but not beta-catenin, mutation represents an early event in rat hepatocarcinogenesis. Hepatology.

[6] Chaiteerakij R, Addissie BD, Roberts LR (2015). Update on biomarkers of hepatocellular carcinoma. Clin Gastroenterol Hepatol.

[7] Jin S, Wang K, Xu K, Xu J, Sun J, Chu Z, Lin D, Koeffler PH, Wang J, Yin D (2014). Oncogenic function and prognostic significance of protein tyrosine phosphatase PRL-1 in hepatocellular carcinoma. Oncotarget.

[8] Friemel J, Rechsteiner M, Frick L, Bohm F, Struckmann K, Egger M, Moch H, Heikenwalder M, Weber A (2015). Intratumor heterogeneity in hepatocellular carcinoma. Clin Cancer Res.

[9] Bruix J, Gores GJ, Mazzaferro V (2014). Hepatocellular carcinoma: clinical frontiers and perspectives. Gut.

[10] Yang J, Li J, Dai W, Wang F, Shen M, Chen K, Cheng P, Zhang Y, Wang C, Zhu R, Zhang H, Zheng Y, Wang J (2015). Golgi protein 73 as a biomarker for hepatocellular carcinoma: A diagnostic meta-analysis. Exp Ther Med.

[11] Luo Q, Zhang Y, Wang N, Jin G, Jin H, Gu D, Tao X, Huo X, Ge T, Cong W, Wang C, Qin W (2015). Leukemia inhibitory factor receptor is a novel immunomarker in distinction of well-differentiated HCC from dysplastic nodules. Oncotarget.

[12] Nikolov M, Schmidt C, Urlaub H (2012). Quantitative mass spectrometry-based proteomics: an overview. Methods Mol Biol.

[13] Xu D, Li Y, Li X, Wei LL, Pan Z, Jiang TT, Chen ZL, Wang C, Cao WM, Zhang X, Ping ZP, Liu CM, Liu JY (2015). Serum protein S100A9, SOD3, and MMP9 as new diagnostic biomarkers for pulmonary tuberculosis by iTRAQ-coupled two-dimensional LC-MS/MS. Proteomics.

[14] Cai MY, Tong ZT, Zheng F, Liao YJ, Wang Y, Rao HL, Chen YC, Wu QL, Liu YH, Guan XY, Lin MC, Zeng YX, Kung HF (2011). EZH2 protein: a promising immunomarker for the detection of hepatocellular carcinomas in liver needle biopsies. Gut.

[15] Tremosini S, Forner A, Boix L, Vilana R, Bianchi L, Reig M, Rimola J, Rodriguez-Lope C, Ayuso C, Sole M, Bruix J (2012). Prospective validation of an immunohistochemical panel (glypican 3, heat shock protein 70 and glutamine synthetase) in liver biopsies for diagnosis of very early hepatocellular carcinoma. Gut.

[16] Di Tommaso L, Destro A, Seok JY, Balladore E, Terracciano L, Sangiovanni A, Iavarone M, Colombo M, Jang JJ, Yu E, Jin SY, Morenghi E, Park YN (2009). The application of markers (HSP70 GPC3 and GS) in liver biopsies is useful for detection of hepatocellular carcinoma. J Hepatol.

[17] Cheng J, Xie HY, Xu X, Wu J, Wei X, Su R, Zhang W, Lv Z, Zheng S, Zhou L (2011). NDRG1 as a biomarker for metastasis, recurrence and of poor prognosis in hepatocellular carcinoma. Cancer Lett.

[18] Lu WJ, Chua MS, So SK (2014). Suppressing N-Myc downstream regulated gene 1 reactivates senescence signaling and inhibits tumor growth in hepatocellular carcinoma. Carcinogenesis.

[19] Akiba J, Murakami Y, Noda M, Watari K, Ogasawara S, Yoshida T, Kawahara A, Sanada S, Yasumoto M, Yamaguchi R, Kage M, Kuwano M, Ono M (2011). N-myc downstream regulated gene1/Cap43 overexpression suppresses tumor growth by hepatic cancer cells through cell cycle arrest at the G0/G1 phase. Cancer Lett.

[20] Zhang Y, Li Y, Lin C, Ding J, Liao G, Tang B (2014). Aberrant upregulation of 14-3-3sigma and EZH2 expression serves as an inferior prognostic biomarker for hepatocellular carcinoma. PLoS One.

[21] Gheibi A, Kazemi M, Baradaran A, Akbari M, Salehi M (2012). Study of promoter methylation pattern of 14-3-3 sigma gene in normal and cancerous tissue of breast: A potential biomarker for detection of breast cancer in patients. Adv Biomed Res.

[22] Qi YJ, Wang M, Liu RM, Wei H, Chao WX, Zhang T, Lou Q, Li XM, Ma J, Zhu H, Yang ZH, Liu HQ, Ma YF (2014). Downregulation of 14-3-3sigma correlates with multistage carcinogenesis and poor prognosis of esophageal squamous cell carcinoma. PLoS One.

[23] Han G, Fan M, Zhang X (2015). microRNA-218 inhibits prostate cancer cell growth and promotes apoptosis by repressing TPD52 expression. Biochem Biophys Res Commun.

[24] Wang Z, Sun J, Zhao Y, Guo W, Lv K, Zhang Q (2014). Lentivirus-mediated knockdown of tumor protein D52-like 2 inhibits glioma cell proliferation. Cell Mol Biol (Noisy-le-grand).

[25] Roslan N, Bieche I, Bright RK, Lidereau R, Chen Y, Byrne JA (2014). TPD52 represents a survival factor in ERBB2-amplified breast cancer cells. Mol Carcinog.

[26] Goto Y, Nishikawa R, Kojima S, Chiyomaru T, Enokida H, Inoguchi S, Kinoshita T, Fuse M, Sakamoto S, Nakagawa M, Naya Y, Ichikawa T, Seki N (2014). Tumour-suppressive microRNA-224 inhibits cancer cell migration and invasion via targeting oncogenic TPD52 in prostate cancer. FEBS Lett.

[27] Ko HL, Ren EC (2011). Novel poly (ADP-ribose) polymerase 1 binding motif in hepatitis B virus core promoter impairs DNA damage repair. Hepatology.

[28] Dal Bello B, Rosa L, Campanini N, Tinelli C, Torello Viera F, D'Ambrosio G, Rossi S, Silini EM (2010). Glutamine synthetase immunostaining correlates with pathologic features of hepatocellular carcinoma and better survival after radiofrequency thermal ablation. Clin Cancer Res.

[29] Osada T, Nagashima I, Tsuno NH, Kitayama J, Nagawa H (2000). Prognostic significance of glutamine synthetase expression in unifocal advanced hepatocellular carcinoma. J Hepatol.

[30] Huang C, Wang Y, Liu S, Ding G, Liu W, Zhou J, Kuang M, Ji Y, Kondo T, Fan J (2013). Quantitative Proteomic Analysis Identified Paraoxonase 1 as a Novel Serum Biomarker for Microvascular Invasion in Hepatocellular Carcinoma. J Proteome Res.

