# Supplementary material for: Global proteomic profiling in multistep hepatocarcinogenesis and identification of PARP1 as a novel molecular marker in hepatocellular carcinoma
Source: Oncotarget. 2016 Feb 11;7(12):13730–41. doi: 10.18632/oncotarget.7316 (PMC4924674; doi:10.18632/oncotarget.7316)
Supplement: Supplementary file 1 [file oncotarget-07-13730-s001.pdf]

Global proteomic profiling in multistep hepatocarcinogenesis and identification of PARP1 as a novel molecular marker in hepatocellular carcinoma

Supplementary Materials

Supplementary Table S1: Clinical and demographic characteristics of subjects in Cohort 1

Variables	NL	HL	CL	PL	HCC
	(*n* = 15)	(*n* = 15)	(*n* = 15)	(*n* = 15)	(*n* = 15)
Median age (quartile)	48 (40–54)	54 (45–60)	51 (49–59)	53 (50–56)	53 (50–56)
Sex (M/F)	8/7	9/6	10/5	11/4	11/4
HBsAg (N/P)	15/0	0/15	0/15	0/15	0/15
Tumor size (cm)	NA	NA	NA	NA	1.4 ± 0.4
Tumor (S/M)	NA	NA	NA	NA	15/0

Abbreviations: normal livers, NL; hepatitis livers, HL; cirrhotic livers, CL; peritumoral livers, PL; hepatocellular carcinoma livers, HCC; HBsAg, hepatitis B surface antigen; Sex, M/F denotes male/female; HBsAg, N/P denotes negative/positive; Tumor number, S/M denotes single/multiple; NA, not applied.

Supplementary Table S2: List of 37874 peptides identified in HCC samples by iTRAQ

Supplementary Table S3: List of 3017 Proteins identified in HCC samples by itraq. NL, 113; HL, 114; CL, 116; PL, 119; HCC, 121

Supplementary Table S4: Clinical and demographic characteristics of subjects in Cohort 2

Variables	No. (%) of Patients					*p*-Value*
	NL (*n* = 7)	CL (*n* = 19)	DN (*n* = 14)	HCC (*n* = 51)	ICC (*n* = 23)	
Age						0.067
≤ 50	5 (71.4)	14 (73.1)	5 (35.7)	26(51.0)	8 (34.8)	
> 50	2 (28.6)	5 (26.3)	9 (64.3)	25(49.1)	15 (65.2)	
Sex						0.022
Male	5 (71.4)	12 (63.2)	10 (71.4)	43(84.3)	11 (47.8)	
Female	2 (28.6)	7 (36.8)	4 (28.6)	8(15.7)	12 (52.2)	
HBsAg						< 0.001
Positive	0 (0)	19 (100)	14 (100)	51(100)	23 (100)	
Negative	7 (100)	0 (0)	0 (0)	0(0)	0 (0)	
Tumor size						0.106
≤ 5 cm	NA	NA	NA	38 (74.5)	12 (52.2)	
> 5 cm	NA	NA	NA	13 (25.5)	11 (47.8)	
Grading						
G1/G2	NA	NA	NA	24 (47.1)	10 (43.5)	0.806
G3	NA	NA	NA	27 (52.9)	13 (56.5)	

Abbreviations: normal livers, NL; cirrhotic livers, CL; dysplastic nodules, DN; hepatocellular carcinoma livers, HCC; HBsAg, hepatitis B surface antigen; NA, not applied. **P*-values were calculated using the Pearson chi-square test.

Supplementary Table S5: Clinical and demographic characteristics of HCC patients in Cohort 3

Variables	HCC (*n* = 180)
**Age**	
≤ 50	62(34.4)
> 50	118(65.6)
**Sex**	
Male	29(716.1)
Female	151(83.9)
**HBsAg**	
Positive	180(100)
Negative	0(0)
**Tumor size**	
≤ 5 cm	75(41.7)
> 5 cm	105(58.3)
**Tumor multiplicity**	
Single	155(86.1)
Multiple	25(13.9)
**Differentiation**	
Well	23(12.8)
Moderate	98(54.4)
Poor	59(32.8)
**TNM stage**	
I	23(12.8)
II	76(42.2)
III	71(39.4)
IV	10(5.6)

Supplementary Table S6: The antibodies and dilution used in this study

	14-3-3sigma	NDRG1	TPD52	FDPS	GS	PARP1	HSP70
Company	Abcam	Abcam	Abcam	Abcam	Abcam	Abcam	Abcam
Name	Anti-14-3-3 sigma	Anti-NDRG1	Anti-TPD52	Anti-FDPS	Anti-Glutamine Synthetase	Anti-PARP1	Anti-HSP70
Dilution	1:50	1:300	1:100	1:200	1:200	1:150	1:400
